# Cognitive Challenge to Choose Healthier Food Is Reflected in Heart Rate Variability

**DOI:** 10.3389/fnins.2017.00353

**Published:** 2017-06-23

**Authors:** Maryam Haghshomar, Farzaneh Rahmani

**Affiliations:** ^1^Students Scientific Research Center, Tehran University of Medical SciencesTehran, Iran; ^2^NeuroImaging Network, Universal Scientific Education and Research Network (USERN)Tehran, Iran

**Keywords:** food choice, prefrontal cortex, health knowledge, attitudes, practice, ventromedial prefrontal cortex, dorsolateral prefrontal cortex (DLPFC), fMRI BOLD

Choice of healthier over tastier food is the ever existing challenge of the human being. This choice reflects a complex decision-making circuit in the mammalian brain that validates and interprets the value of external stimuli in a multi-attribute model. Food choice is a goal-directed decision that enroots in the value signal attributed to each item by the ventromedial prefrontal cortex (vmPFC), which is part of brain valuation and reward circuits. Herein we present results of a recent research paper by Maier et al. (Maier and Hare, 2017) in an analytical view, comparing it to two earlier published articles by DelParigi et al. (2007) and Hare et al. (2009).

DelParigi et al. (2007) were the first group to reveal that cortical areas of the reward system activate during food choice dilemmas and that the dorsolateral prefrontal cortex (dlPFC) is more active in successful dieters. They utilized the 51-item Three-Factor Eating Questionnaire (TFEQ) to measure dietary restraint, dietary disinhibition, and hunger and positron emission tomography with (15)O water to track brain areas with increased activity during satiety and fasting.

DelParigi et al. (2007) sought to identify brain areas with increased activity in successful dieters. Interestingly, they found that successful dieters were not any different in their perception of hunger, vulnerability to dietary disinhibition or their hedonic response to food taste from their peer control group. This control group were those who were not on any weight loss program and had not experienced weight change over a period of 3 months (DelParigi et al., 2007).

While dietary self-control success (SCS) showed association with better scores in dietary restraint in the TFEQ, DelParigi did not find any association between behavioral scores of dietary restraint with changes in brain activity on PET scan. Nonetheless, they found a positive correlation between the neural activity in the dlPFC area with the scores subjects gave to sweetness, creaminess, and pleasantness of food items in a “taste preference” experiment. They found a parallel decrease in activity at the orbitofrontal cortex (OFC) on PET scans, an associational brain area with visceral and sensory inputs. These results supported the two valuation system view to decision making in which the dlPFC enforces a top-down control over other frontal cortical areas.

One major pitfall to this study was the block design of the study and sequential measurement of brain activity, i.e., the PET maps of neural activity were obtained after 36 h of fasting following the dietary restraint tests. This issue was later considered in the studies that followed by Hare et al. (2009) and recently by Maier et al. (Maier and Hare, 2017).

Hare et al. investigated this hypothesis in 2009 (Hare et al., 2009) through BOLD fMRI imaging. BOLD fMRI is a direct measurement of brain activity and gives higher spatial specificity when compared to measuring regional changes in cerebral blood flow (rCBF), used by DelParigi et al. (2007).

One advantage of Hare et al.'s experiment was that they identified a “reference object” for each participant based on their self-declared health/taste value. This enabled them to attribute an integrated, unique score (goal value) of the overall health and taste reward each participant gained by choosing each food item. They demonstrated through generalized linear model regression that it was only the activity of vmPFC that correlated with the integrated goal values. On the contrary, the dlPFC in the inferior frontal gyrus was only active during trials in which the overall healthier object was preferred over the overall tastier one. Results of this study were in favor of the existence of a unique valuation system that resides in the vmPFC. Of interest, Hare and his colleagues failed to demonstrate functional connectivity between the dlPFC and vmPFC areas.

We now focus on the results from the latest study by Maier et al. (Maier and Hare, 2017). Maier and her colleagues tried to model dietary self-control ability by integrating the following variables: (1) self-rated scales of health and taste status of the food objects, (2) their self-reported ability in dietary control in TFEQ, (3) stress as a confounding factor in decision making, and finally (4) heart rate variability (HRV). They investigated whether HRV could be used as a proxy measurement of psychophysiological reactivity to social stress (van Hedger et al., 2017) and self-regulatory abilities (Fagan et al., 2017).

Similar to Hare et al. they ran a validation experiment to identify health/taste value for each food object. As a first step, they assessed how much participants valued health, taste and the appetizing features of each food on an 180-item series of food objects. Individuals had to rate items on a visual scale based on their perception of their relative health or taste. Different from Hare et al. they attributed an absolute, not relative, value to health and taste of food items. This subjective measurement of values gave them the opportunity to calculate an overall mean difference in health (Hdiff) and taste (Tdiff) in the trial for each participant.

One significant experimental advantage of the study by Maier was that only half of the trials included dietary challenges i.e., health and taste conflict. Meanwhile, they had instructed participants to choose the healthier object which occasionally happened to be the tastier one as well. This additional measure enabled them to control for response-set bias, i.e., turning down the tastier object in all trials because it appears to be the right choice according to previous questions, which was not controlled in previous studies.

It is known that stress and anxiety can adversely affect attention and executive functions. Stress prevents cognitive control over distracting stimuli during executive tasks (Ramírez et al., 2015) and impairs working memory (Laborde et al., 2015). HRV is a common proxy measure to assess autonomic as well as peripheral nervous system balance and function. HRV might be able to represent levels of social stress and emotional instability in decision making (Fagan et al., 2017), psychophysiological reactivity (van Hedger et al., 2017), and self-regulatory abilities such as decision-making (Fagan et al., 2017).

There is a direct relation between the level of anxiety, low variance in heart rate (low HRV) and decreased ability in cognitive control of attention and risk assessment in decision making (Ramírez et al., 2015). HRV is perhaps a reflection of an autonomous system endeavor to regulate physiological responses during the ever existing challenges of adapting to distracting or dangerous stimuli. Maier and his colleagues addressed the relationship between dietary self-control, as a complex emotional and subcortical decision-making process, and HRV using fMRI tools (Maier and Hare, 2017). Imaging data indicates that higher HRV correlates with decreased representation of taste attributes in brain regions regulating autonomic responses and decision making, i.e., vmPFC.

Central nervous system substrates that control heart rate in the mammalian brain can be divided into two main clusters: The “central autonomous network” (CAN) and critical and subcortical projections that regulate the CAN (Duggento and Bianciardi, 2016). Neural substrates of the first system that directly controls autonomous function are areas in the amygdala, hypothalamus, and brainstem (Wheelock et al., 2016). Cortical afferents/efferents to and from prefrontal cortex (PFC), as well as superior, middle and inferior frontal gyrus and areas in the parietal cortex, comprise the second network (Meeten et al., 2016).

Maier et al. calculated the two common measures of HRV: First, they calculated at-rest measurements of the standard deviation of all RR intervals (R wave to R wave interval) i.e., the SDNN (SDNN Standard deviation of normal RR intervals). Second, they used the “root mean square of successive differences” (RMSSD). Maier et al. chose SDNN values to build the dietary self-control ability model, as it better reflects the overall confounding effect of external factors that alter cognitive control of food choice.

Maier et al. demonstrated through their interesting experiments, that HRV is a robust predictor of dietary self-control, either in terms of self-control trait in the TFEQ i.e., restrained eating (RE) or in dietary challenges i.e., SCS. Stress was able to mitigate cognitive control over food choice but unable to disrupt HRV and RE correlation with SCS. RE and HRV together predicted 77% of the overall variance of SCS.

Maier et al. next implemented BOLD modality using fMRI to identify neuronal clusters that correlated with HRV during food choice challenges. Comparable to findings of Hare et al. (2009) the functional activity of vmPFC in food choice challenge trials, could predict resting HRV of individuals. As a final step to confirm the value-attributing function of the vmPFC, they investigated whether cortical activity in vmPFC could rightfully reflect the integrated calculated value of food items, only to find a significant correlation between encoded signals in this region of interest (ROI) and the integrated health/taste score of the food objects.

Eating behavior is a complex decision-making process. The vmPFC and dlPFC are main areas involved in complex decision-making and self-control. vmPFC attributes a value to the short-term significance of a choice, which in the case of a food item is a measurement of its taste. Then, the dlPFC contributes to the final decision by attributing a long-term value to the choice i.e., health of the food item.

Through their exquisitely designed experiment, Maier and her colleagues show that humans' ability in dietary self-control can be predicted in a model of self-declaration of restrained eating ability, HRV, and self-perception of health/taste values of food objects. They confirmed through BOLD fMRI that the calculated integrated score of dietary self-control from this model strongly predicts the degree of vmPFC activity during food-choice dilemmas.

## Author contributions

MH has reviewed the literature and drafted the article. FR made critical reviews and added expert opinion to the draft.

### Conflict of interest statement

The authors declare that the research was conducted in the absence of any commercial or financial relationships that could be construed as a potential conflict of interest.
